# Idiopathic ventricular fibrillation: is it a case for genetic testing?

**DOI:** 10.1007/s00399-024-00994-3

**Published:** 2024-02-09

**Authors:** S. N. van der Crabben, A. A. M. Wilde

**Affiliations:** 1 Department of Human Genetics, Amsterdam UMC, University of Amsterdam, Meibergdreef 9, 1105 AZ Amsterdam, The Netherlands; 2 European Reference Network for rare, low prevalence, and/or complex diseases of the heart: ERN GUARD-Heart, Amsterdam, The Netherlands; 3 Amsterdam UMC, Department of Cardiology, University of Amsterdam, Meibergdreef 9, Amsterdam, The Netherlands; 4 Amsterdam Cardiovascular Sciences, Heart Failure and arrhythmias, Amsterdam, The Netherlands

**Keywords:** IVF, DNA, Deep phenotyping, Inherited cardiac condition, Sudden cardiac death, Idiopathisches Kammerflimmern, DNA, Umfassende Phänotypisierung, Erbliche Herzerkrankung, Plötzlicher Herztod

## Abstract

Idiopathic ventricular fibrillation (IVF) is a diagnosis of exclusion in sudden cardiac arrest (SCA) survivors. Although there are clear guidelines on the clinical work-up of SCA survivors, less than one in five patients receives a complete work-up. This increases the chances of erroneously labelling these patients as having IVF, while 10–20% of them have an inherited cardiac condition (ICC). Diagnoses of ICC increase over time due to (additional) deep phenotyping or as a result of spontaneous expression of ICC over time. As SCA survivors can also harbor (likely) pathogenic variants in cardiomyopathy-associated genes in the absence of a phenotype, or can have another ICC without a clear cardiac phenotype, the question arises as to whether genetic testing in this group should be routinely performed. Family history (mainly in the case of sudden death) can increase suspicion of an ICC in an SCA victim, but does not add great value when adults underwent a complete cardiological work-up. The diagnosis of ICC has treatment consequences not only for the patient but also for their family. Genetic diagnostic yield does not appear to increase with larger gene panels, but variants of unknown significance (VUS) do. Although VUS can be confusing, careful and critical segregation analysis in the family can be performed when discussed in a multidisciplinary team at a center of expertise with at least a cardiologist as well as a clinical and laboratory geneticist, thereby degrading or promoting VUS. When to introduce genetic testing in SCA survivors remains a matter of debate, but the combination of quick, deep phenotyping with additional genetic testing for the unidentifiable phenotypes, especially in the young, seems preferable.

## Abstract (de)

Idiopathisches Kammerflimmern („idiopathic ventricular fibrillation“ [IVF]) ist eine Ausschlussdiagnose bei Überlebenden eines plötzlichen Herzstillstands („sudden cardiac arrest“ [SCA]). Obwohl es eindeutige Leitlinien für die klinische Abklärung von SCA-Überlebenden gibt, erhält weniger als einer von fünf betroffenen Patienten eine vollständige Diagnostik. Dadurch erhöht sich das Risiko einer falschen Diagnose von IVF bei diesen Patienten, von denen 10–20% eine erbliche Herzerkrankung („inherited cardiac condition“ [ICC]) haben. ICC-Diagnosen nehmen mit der Zeit zu, bedingt durch (zusätzliche) umfassende Phänotypisierung oder als Ergebnis einer spontanen ICC-Manifestation im Verlauf. Da SCA-Überlebende auch Träger (wahrscheinlich) pathogener Varianten kardiomyopathieassoziierter Gene bei Fehlen eines Phänotyps sein können oder eine andere ICC ohne eindeutigen kardialen Phänotyp aufweisen können, stellt sich die Frage, ob in dieser Patientengruppe eine genetische Diagnostik routinemäßig durchgeführt werden sollte. Die Familienanamnese (hauptsächlich bei plötzlichem Todesfall) kann den Verdacht auf eine ICC bei Patienten mit SCA erhärten, hat aber keinen hohen zusätzlichen Wert, wenn Erwachsene einer vollständigen kardiologischen Abklärung unterzogen worden sind. Die Diagnose einer ICC hat nicht nur für den Patienten, sondern auch für dessen Familie therapeutische Konsequenzen. Die gendiagnostische Ausbeute scheint sich mit größeren Genpanels nicht zu erhöhen, anders verhält es sich in Bezug auf Varianten mit ungeklärter Relevanz („variants of unknown significance“ [VUS]). Auch wenn VUS für Verwirrung sorgen können, kann eine sorgfältige und kritische Segregationsanalyse in der Familie durchgeführt werden, wenn sie in einem multidisziplinären Team an einem spezialisierten Zentrum mit zumindest einem Kardiologen, einem klinischen Genetiker und einem im Laborbereich tätigen Genetiker („laboratory geneticist“) diskutiert wird, woraufhin die VUS hinsichtlich ihrer Bedeutung verworfen oder gestärkt wird. Wann die genetische Diagnostik bei SCA-Überlebenden einbezogen werden soll, ist eine Frage, über die weiter debattiert wird. Die Kombination einer schnellen, umfassenden Phänotypisierung mit zusätzlichen Gentests für die unidentifizierbaren Phänotypen scheint aber zu bevorzugen zu sein, insbesondere bei jungen Patienten.

## Introduction

The diagnosis of idiopathic ventricular fibrillation (IVF) in sudden cardiac arrest (SCA) survivors is reached after exclusion of toxicological compounds and clear underlying structural, electrophysiological or metabolic abnormalities [23]. Genetic testing is mainly indicated in the case of suspicion of inherited cardiac conditions (ICC) after complete cardiological work-up [23, 31]. However, clinical signs of ICC can be absent or missed (due to incomplete/misinterpreted cardiological test results), thereby wrongfully labelling patients as having IVF [4, 5, 9, 16, 29]. Since a diagnosis of ICC in IVF patients influences not only their own but also their family’s therapeutic options, one could argue that genetic testing should be more widely used in IVF patients.

## Idiopathic ventricular fibrillation: is it a time- and clinical evaluation-dependent definition?

SCA survivors receive a broad clinical evaluation including primary cardiac screening (ECG, ultrasound and coronary angiography) to identify the cause of their almost lethal cardiac arrhythmia, including ventricular fibrillation (VF) [23, 31]. This screening reveals a cardiac cause of the arrhythmia in most (~60%) [4, 6] adult SCA survivors. The most frequent underlying cardiac diseases include ischemic heart disease (IHD) in 50–73% of the cases, followed by structural non-ischemic heart disease (12–13%) and primary electrical disease (2–5%) [6, 29]. In the case of structural, non-ischemic heart disease, dilated cardiomyopathy is the most common, followed by hypertrophic and arrhythmogenic cardiomyopathy. In the case of primary electrical cardiac disease, Brugada syndrome is the most common, followed by congenital long QT syndrome (LQTS) and catecholaminergic polymorphic ventricular tachycardia (CPVT) [6, 29]. Clinical diagnosis of these ICCs is a class 1 indication for genetic testing [23].

In a small group of SCA survivors (numbers ranging from 1.2 to 12.3% [4, 29]), primary clinical evaluation does not lead to a clinical diagnosis. In such cases, additional cardiological screening (cardiovascular magnetic resonance imaging [CMR]), high lead and repeated 12-lead ECGs, a sodium channel blocker test and exercise test) is recommended (Class I) [23, 31]. This additional deep phenotyping has been shown to establish a clinical diagnosis in SCA survivors in around 20–44% [2, 16, 29]. CMR and repeated ECG most frequently lead to a clinical change in diagnosis [16, 29], followed by sodium channel blocker tests [29]. Genetic testing, however, was reported to have the highest diagnostic impact [2, 16].

Unfortunately, in daily clinical practice, the required comprehensive clinical work-up of SCA survivors seems to be difficult. Indeed, less than one in five of these patients ultimately received complete cardiological work-up [9, 29], which increases the chance of erroneously labelling those patients as having IVF. However, even when cardiological work-up has been completed, ICC can still be missed due to its rarity.

**Fig. 1:**
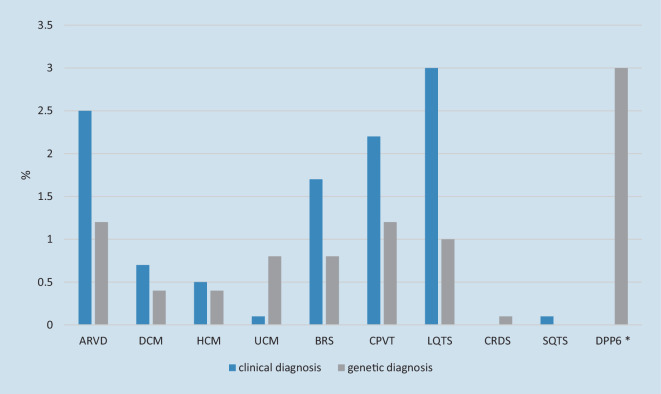
Diagnosis made in idiopathic ventricular fibrillation (*IVF*) (*n* = 1207) patients when followed in time. Percentages were calculated from pooled data of IVF patients previously described [4, 9, 10, 13, 17, 28]. Change in diagnosis was calculated per group (clinical diagnosis and genetic diagnosis) and calculated as a percentage of the whole group of IVF patients *n* = 1207, respectively: *n* = 63 [13], *n* = 107 [28], *n* = 375 [17], *n* = 11 [4], *n* = 228 [10], *n* = 423 (of whom *n* = 367 received genetic testing); NB data from Supplementary Table 3 were used to calculate genetic cause: for arrhythmogenic right ventricular dysplasia (ARVD): *PLN* and *PKP2*, for DCM: *LMNA* and *TTN*, for HCM: *MYH7* and *MYL2*, for Brugada syndrome: *SCN5A*, for CPVT: *RYR2*, for LQTS: *KCNQ1 *[9]. With regard to DPP6*: this data is derived from patients living in a specific region in the Netherlands with high prevalence of this founder variant. *DCM* dilated cardiomyopathy, *HCM* hypertrophic cardiomyopathy, *UCM* unclassified cardiomyopathy, *BRS* Brugada syndrome, *CPVT* catecholaminergic polymorphic ventricular tachycardia, *LQTS* long QT syndrome, *CRDS* calcium release deficiency syndrome, *SQTS* short QT syndrome, Dutch DPP6 haplotype

Clinical re-evaluation and follow-up of SCA survivors diagnosed with IVF in centers of expertise in ICC has proven to lead to a change in diagnosis. ICC can also become apparent over time and be confirmed with genetic testing (Fig. 1; [4, 9, 28]).

However, in some SCA survivors, no phenotype is identified, despite extensive clinical cardiological evaluation. These patients could have a “concealed” ICC with (likely) pathogenic genetic variants in one of the cardiomyopathy-associated genes [11]. Alternatively, these patients might have (likely) pathogenic variants in genes associated with VF without cardiac phenotype such as: *CALM1 *[15], *IRX3 *[12], the more recently identified loss-of-function *RYR2* variants associated with calcium-release deficiency syndrome (CRDS) [22] or the Dutch *DPP6* haplotype [1]. In a Dutch IVF cohort, the latter was shown to be the cause in 10% of genetically tested patients [9]. Importantly, when cardiological screening does not provide clues for the diagnosis of ICC in the patient, analysis of the family can be of help.

## SCA survivors and family screening for ICC

When considering ICC, a detailed patient history, including the presence of previous symptoms and the circumstances of the cardiac arrest (e.g. rest or exercise), is important [30]. Proper family history-taking is crucial, as other family members might have similar features, a more prominent phenotype or even a diagnosis of ICC. Therefore, clinical evaluation and cardiological screening of family members is advised in the work-up of IVF patients [23]. A positive family history, mainly in the case of sudden death (SD), increases the chance for the patient to have ICC [9, 17]. However, the yield of additional family screening after thorough clinical investigation of the SCA survivor is very low (3%) [18]. In the case of an ICC without a clear recognizable phenotype, such as the Dutch *DPP6* haplotype, family screening will not be helpful in achieving a clinical diagnosis of ICC, as affected family members show no clinical abnormalities at extensive cardiological screening [1].

The low diagnostic yield of family screening in SCA survivors seems in contrast with the yield of family screening after SD [14]. However, in the case of aborted SD, deep phenotyping of the index case (e.g. ECG, exercise test) is available and will reveal the underlying cause in the vast majority of cases, making family screening for diagnostic purposes unnecessary.

## Genetic testing in SCA survivors

In general clinical care, there is a growing tendency to perform genetic testing at an early stage in critically ill patients without a clear clinical diagnosis, firstly via whole exome sequencing. Indeed, the introduction of rapid exome sequencing in critically ill neonates has proven to increase diagnostic yield, reduce time to diagnosis and decrease healthcare costs [20]. In SCA survivors, the indication for and timing of genetic testing will differ between individual patients.

In SCA survivors with clinical suspicion of an ICC after first cardiological evaluation, targeted genetic testing can be directly initiated [23]. For other SCA survivors, genetic testing will be performed when, after additional cardiac phenotyping, suspicion of ICC is substantiated [9]. However, the timeframe within which additional investigations in SCA survivors without a clinical diagnosis should be performed is not clearly defined and therefore can cause significant delay. Other reasons why additional deep phenotyping may be hampered is for example physical inability to perform an exercise test. In such cases, missing out relevant data can simply mean missing the clinical diagnosis of ICC.

Nevertheless, although combining deep phenotyping with whole exome sequencing (WES) in SCA survivors nearly doubles the diagnosis of ICC to 20%, half of the diagnoses contained (likely) pathogenic variants in cardiomyopathy-associated genes in patients without clinical signs of cardiomyopathy [10]. Therefore, the timing of genetic testing in SCA survivors can be debated, as a genetic diagnosis can potentially have an immediate impact on the patient who survived a cardiac arrest episode.

With the diagnosis of IVF, implantable cardioverter-defibrillator (ICD) implantation usually follows [31]. However, additional pharmacological treatment is pertinent in the case of specific primary arrhythmia syndromes, and occasionally one may even refrain from ICD implantation. Patients with CPVT may benefit from ICD treatment, but there is the potential for proarrhythmia with lethal consequences [26]. Therefore, ICD implantation in resuscitated patients, unknown to have CPVT and therefore untreated, should not be automatic. Indeed, in a relatively large retrospective study, ICD treatment in resuscitated CPVT patients was not shown to improve survival [25]. As an exercise test is frequently lacking in the initial investigations after a cardiac arrest [7], presumably often for logistical reasons, the diagnosis of CPVT will be missed. With early genetic testing (and a rapid turnaround of the results), the correct diagnosis will be reached earlier. However, the authors emphasize the importance of a complete cardiological investigation before genetic testing is initiated. Especially in the case of finding a variant of unknown significance (VUS), complete clinical work-up is crucial for correct interpretation. Given its great importance, the authors advise that an additional cardiac work-up be performed as soon as possible after resuscitation (ideally within a few weeks) in order to prevent a diagnostic delay. In SCA survivors without a clinical diagnosis and unable to perform an exercise test as a result of, for instance, anoxic damage, early and rapid genetic testing may be a good option.

Aside from the direct therapeutic consequences for the patient, the diagnosis of ICC also greatly affects morbidity and mortality in the patient’s family [13, 19]. In diseases such as the Dutch *DPP6* haplotype, identification of family members at risk and primary prevention ICD implantation has saved lives [3]. However, also in other ICCs, such as LQTS or Brugada syndrome, lifestyle advice or the avoidance of specific drugs will lower the morbidity and mortality rate of everyone involved.

In general, genetic testing in IVF patients identifies a (likely) pathogenic DNA variant in ~6–10% of these patients [17, 21, 28]. Numbers can increase dramatically when SCA survivors are younger [2, 8]. The type of genetic test ordered can vary: 1) a targeted gene test, for instance in the case of strong clinical suspicion of a specific ICC; 2) a gene panel (varying from small to larger gene panels); and 3) WES in the case of a broad (research) analysis.

Increasing the numbers of genes analyzed will increase the number of VUS without increasing the chance of identifying (likely) pathogenic variants [10, 27]. VUS can create a lot of “fuzz” not only for doctors but also for patients when they are informed about them [24]. However, the question remains as to what the true meaning of a VUS is, especially since correlation with the likelihood of ventricular arrhythmia recurrence has been noted [21], and ultimately, VUS may turn out to be pathogenic [17].

Classifying a genetic variant depends on: 1) the rarity of the gene, 2) the rarity of the specific variant in patients or control cohorts and 3) the availability of or possibility for functional testing [19]. A VUS means that at the moment of classification, there is too little evidence to link the specific genetic variant to the disease. An important side note is that available information between genetic laboratories can differ, affecting the (local) classification of the genetic variant.

However, the clinical presentation of the patient combined with their family history can change the interpretation of VUS into suspicious for pathogenicity (“hot”) [17]. It is therefore of the utmost importance to carefully discuss and weigh the meaning of VUS (hot or not?) after complete cardiological work-up (if possible) in a multidisciplinary setting with at least a (pediatric) cardiologist as well as a clinical and laboratory geneticist [23, 24]. The authors previously proposed that only in the case of suspicion of pathogenicity should “hot VUS” be shared with the patient and segregated in the family [24]. Sharing only hot VUS with SCA patients will both lower unnecessary worries, fears and anxiety and open up the possibility of further proving pathogenicity through careful segregation analysis within the family. Data from segregation analysis is needed to demote or promote hot VUS [24]. Careful counseling of the patient and their family, actively collecting clinical data, including a detailed third-degree pedigree, and deep phenotyping are mandatory elements in this process. In the absence of this coordinated setting, VUS will remain VUS. Confusion will remain and family cascade screening cannot be initiated, potentially leaving family members at risk, undiagnosed and untreated.

## Timing and content of genetic testing in SCA survivors

**Fig. 2:**
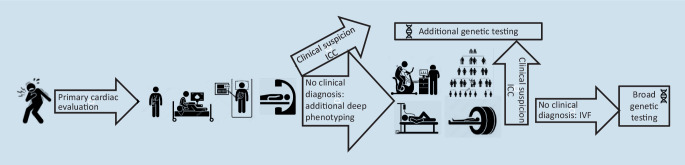
Diagnostic work-up of sudden cardiac arrest survivors and timing of genetic testing. *ICC* idiopathic ventricular fibrillation, *IVF* inherited cardiac condition

In summary, when looking at SCA survivors, there are probably three time points at which genetic testing can be initiated (Fig. 2):

1. When ICC is suspected after *initial clinical cardiac phenotyping*, targeted genetic testing can be ordered (per gene or a small gene panel).
2. When suspicion of ICC has risen after *additional screening* (including detailed family history, with a third-degree pedigree and request for additional family information if indicated) and *deep cardiac phenotyping*, targeted genetic testing can be ordered (per gene or a small gene panel).
3. When the *clinical diagnosis of IVF* is reached despite deep phenotyping, or in the case of inability to perform deep phenotyping in the patient, broader gene panel testing can be considered, especially in younger patients (< 55 years of age). This should be performed preferably at expert centers within the setting of a multidisciplinary team.

Depending on the country of residency of the patient and turnaround time of the genetic laboratory, the results of genetic testing will take several weeks to months. The type of test that can be performed also strongly depends on the possibilities of the genetic laboratory, which differ from country to country. In addition, differences in insurances influence to what extent genetic testing can be performed. With regard to the third option, that is, a broader gene panel in a patient with the clinical diagnosis of IVF, the authors would recommend a panel with at least the genes shown in Tab. 1.

**Table 1:** Proposed gene panel for sudden cardiac arrest survivors with a clinical diagnosis of idiopathic ventricular fibrillation (IVF)

Inherited cardiac condition (ICC)	Associated genes	Proposed IVF gene panel (expert opinion)
LQTS	*CACNA1C, CALM1, CALM2, CALM3, KCNE1, KCNE2, KCNH2, KCNJ2, KCNQ1, SCN5A, TRDN*	Arrhythmia-associated genes *CACNA1C, CALM1, CALM2, CALM3, CASQ2, KCNE1, KCNE2, KCNH2, KCNJ2, KCNQ1, RYR2, SCN5A*
SQTS	*KCNH2, KCNJ2, KCNQ1, SLC4A3*	
CPVT	*CALM1, CALM2, CALM3, CASQ2, KCNJ2, RYR2, TECRL, TRDN*	
BRS	*SCN5A*	
ARVD	*DES, DSC2, DSG2, DSP, JUP, LMNA, PKP2, PLN, TMEM43*	Cardiomyopathy-associated genes *ACTN2, BAG3, DES, DSC2, DSG2, DSP, FLNC, JUP, LMNA, MYBPC3, MYH7, MYL2, MYL3, MYL4, PKP2, PLN, PRKAG2, RBM20, TNNI3, TNNI3K, TTN, TNNT2*
DCM	*BAG3, DES, FLNC, LMNA, MYH7, PLN, RBM20, SCN5A, TNNC1, TNNT2, TTN, TNNI3K*	
HCM	*MYBPC3, MYH7, TNNT2, TNNI3, ACTC1, TPM1, MYL2, MYL3, TNNC1*	
Absent cardiac phenotype	*CALM1, RYR2, IRX3, DPP6*	Genes without discernible cardiac phenotype *IRX3, DPP6*

*LQTS* long QT syndrome, *SQTS* short QT syndrome, *CPVT* catecholaminergic polymorphic ventricular tachycardia, *BRS* Brugada syndrome, *ARVD* arrhythmogenic right ventricular dysplasia, *DCM* dilated cardiomyopathy, *HCM* hypertrophic cardiomyopathy

## Practical conclusions

IVF is a diagnosis of exclusion in SCA survivors that can only be made after complete and deep phenotyping including detailed personal and family history (with pedigree up to third-degree relatives). In the case of clinical suspicion of ICC after complete diagnostic work-up, the authors advise targeted gene analysis or a small gene panel. In the case of inability to perform a complete diagnostic work-up or if there is high clinical suspicion of ICC in an SCA survivor, genetic testing should be carried out, preferably with what is referred to as an IVF gene panel. This gene panel includes all relevant genes related to cardiomyopathy or arrhythmia as well as genes associated with no discernable phenotype. Genetic testing should be initiated and guided by a multidisciplinary team at a center of expertise. Family screening seems mainly useful in cases of suspicious VUS. The timing of genetic testing, bearing in mind therapeutic consequences for the patient and their family, is a matter of debate. Extended longitudinal IVF follow-up studies are needed to reveal the best time point and value of genetic testing and to identify the prevalence of true IVF. Based on current data, the authors argue for at least a consideration of (rare) ICC early on in the differential diagnostic work-up of SCA survivors as well as for the use of genetic testing, sometimes more widely than currently described.

## Declarations

For this article no studies with human participants or animals were performed by any of the authors. All studies mentioned were in accordance with the ethical standards indicated in each case.
